# Anterior Hypopituitarism and Treatment Response in Hunter Syndrome: A Comparison of Two Patients

**DOI:** 10.1155/2016/4328492

**Published:** 2016-11-28

**Authors:** Munier A. Nour, Paola Luca, David Stephure, Xing-Chang Wei, Aneal Khan

**Affiliations:** ^1^Department of Pediatrics, University of Saskatchewan, Royal University Hospital, 103 Hospital Drive, Saskatoon, SK, Canada; ^2^Section of Pediatric Endocrinology, University of Calgary, Alberta Children's Hospital, 2888 Shaganappi Trail NW, Calgary, AB, Canada; ^3^Department of Diagnostic Imaging, University of Calgary, Alberta Children's Hospital, Calgary, AB, Canada; ^4^Departments of Medical Genetics and Pediatrics, University of Calgary, Alberta Children's Hospital, 2888 Shaganappi Trail NW, Calgary, AB, Canada

## Abstract

Hypopituitarism is a clinically important diagnosis and has not previously been reported in Hunter syndrome. We contrast two cases with anatomic pituitary anomalies: one with anterior panhypopituitarism and the other with intact pituitary function. Patient 1, a 10-year-old boy with Hunter syndrome, was evaluated for poor growth and an ectopic posterior pituitary gland. Endocrine testing revealed growth hormone (GH) deficiency, secondary adrenal insufficiency, and tertiary hypothyroidism. An improvement in growth velocity with hormone replacement (GH, thyroxine, and corticosteroid) was seen; however, final adult height remained compromised. Patient 2, a 13-year-old male with Hunter syndrome, was evaluated for growth failure. He had a large empty sella turcica with posteriorly displaced pituitary. Functional endocrine testing was normal and a trial of GH-treatment yielded no significant effect. Panhypopituitarism associated with pituitary anomalies has not been previously reported in Hunter syndrome and was an incidental finding of significant clinical importance. In the setting of documented anterior hypopituitarism, while hormone replacement improved growth velocity, final height remained impaired. In patient 2 with equivocal GH-testing results, treatment had no effect on linear growth. These cases highlight the importance of careful clinical assessment in Hunter syndrome and that judicious hormone replacement may be indicated in individual cases.

## 1. Introduction

Hunter syndrome is an X-linked mucopolysaccharide storage (MPS) disease due to deficiency of iduronate-2-sulfatase activity caused by mutations in the* IDS* gene (OMIM 309900). Patients typically present in early childhood with short stature, coarse facial features, joint contractures, hepatosplenomegaly, valvular heart defects, and, in some children, developmental delay.

The ubiquitous presence of short stature in Hunter syndrome has generally been considered a feature of the disease rather than secondary to an underlying endocrinopathy. Few reports have evaluated growth hormone (GH) function in Hunter syndrome and among those, the GH axis has typically been found to be intact [1, 2]. The ultimate cause of short stature in most cases of Hunter syndrome remains largely unknown; however, it is believed to involve osseous growth plate disturbances [3]. To our knowledge, there are no reports of hypopituitarism in Hunter syndrome.

In this report we present two male patients with severe short stature and Hunter syndrome evaluated for hypopituitarism following discovery of abnormal pituitary anatomy on cranial MRI.  Case 1 was found to have an ectopic posterior pituitary and anterior panhypopituitarism while Case 2 was found to have a large empty sella with posteriorly displaced pituitary and normal pituitary function. These cases highlight that an endocrinopathy may exist in some patients with Hunter syndrome. Moreover, we demonstrate an improvement in growth velocity in the patient with hypopituitarism following the institution of hormone replacement and a lack of response in the patient with intact pituitary function. Both patients remained significantly compromised in terms of final height.

**Case 1 .**

**Figure 1:**
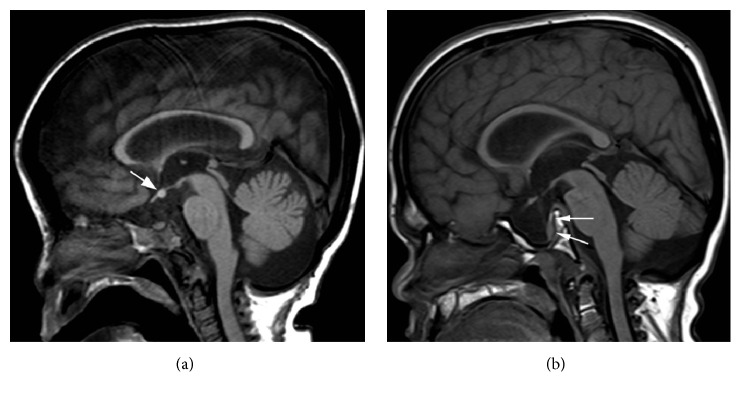
(a) Cranial MRI of Case 1 obtained at the age of 8 years reveals ectopic posterior pituitary lobe. Sagittal spin-echo T1-weighted image shows an abnormal, shallow sella turcica, as well as hypoplastic clivus. A normal T1-hyperintense posterior lobe of the pituitary gland is not identified in the sella turcica. Instead, a T1-hyperintense round structure (arrow) is noted inferior to the floor of third ventricle at the level of infundibular recess. A pituitary stalk is not seen. Prominent forehead related to macrocephaly is also noted. (b) Cranial MRI of Case 2 obtained at the age of 12 years reveals a large empty sella. Sagittal spin-echo T1-weighted image shows a markedly enlarged sella turcica filled with CSF. The anterior and posterior lobes of the pituitary gland (arrows) are displaced posteriorly. A hypoplastic clivus and prominent forehead are present.

This patient presented at 8 months of age with characteristic stigmata of Hunter syndrome. Iduronate 2-sulfatase activity was low (<5 nmol/4 hr/mL plasma; reference 167–475 nmol/4 hr/mL of plasma) (Floyd Snyder, Alberta Children's Hospital, Calgary) and he was found to have a c.213C>A mutation in the* IDS* gene (Peter Ray, The Hospital for Sick Children, Toronto). At 9 years 4 months of age, he was started on intravenous Idursulfase 0.5 mg/kg/week in accordance with standard clinical management of Hunter syndrome [5]. Growth velocity prior to Idursulfase was significantly impaired at 0.7 cm/year (−6.1 S.D.) but improved with treatment to 3.7 cm/year (−2 S.D.). His height at that time was 6.9 S.D. below the mean. At 10 years of age, cranial MRI performed to monitor effacement at the craniocervical junction revealed an ectopic posterior pituitary with a shallow sella turcica (Figure 1(a)). With the exception of severe short stature, symptoms and signs of hypopituitarism were not present.

Given the altered pituitary gland morphology, a complete endocrinologic evaluation demonstrated the following: serum insulin-like growth factor-1 (IGF-1), 58 *μ*g/L (143–693 *μ*g/L; normal for bone age: 50–286 *μ*g/L), early morning serum cortisol, 43 nmol/L (200–690 nmol/L; 1.56 *μ*g/dL (7.25–25 *μ*g/dL)), and free-T4, 7.9 pmol/L (10–25 pmol/L; 0.61 ng/dL (0.78–1.94 ng/dL)). Bone age was 5 years at a chronological age of 11 years.

Peak levodopa/propranolol stimulated serum GH level was 0.2 *μ*g/L at 90 minutes (>8 *μ*g/L). Serum cortisol levels at 0, 30, and 60 minutes following administration of 1 mcg synthetic ACTH (cosyntropin) were 44 (1.59), 149 (5.40), and 177 (6.41) nmol/L (*μ*g/dL), respectively (normal > 500 nmol/L (normal > 18 *μ*g/dL)). Following TRH (protirelin) 0.16 mg intravenously, serum TSH measured 3.15, 28, 45.06, 43.2, and 41.47 mIU/L at 0, 20, 40, 60, and 120 minutes, respectively. These results confirmed GH deficiency, secondary adrenal insufficiency, and tertiary hypothyroidism. Results are summarized in Table 1.

**Figure 2:**
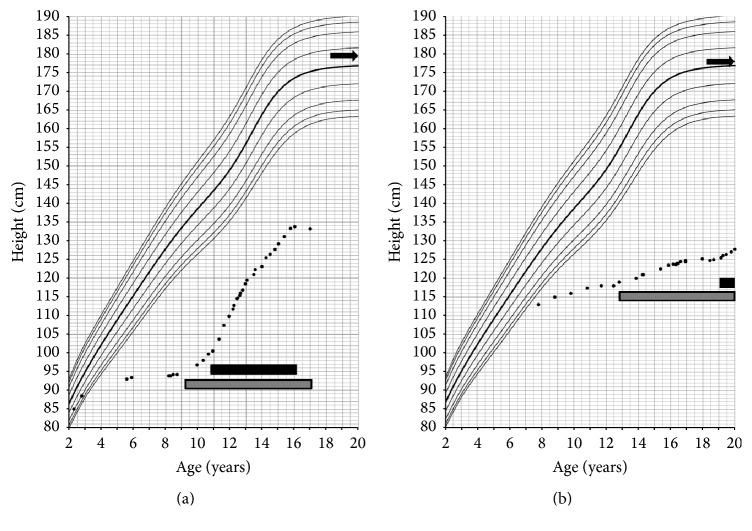
Growth charts of Case 1 (a) and Case 2 (b). Grey bar depicts time of initiation of Idursulfase enzyme replacement and the black bar depicts time of GH treatment. Top arrow depicts calculated midparental target height [4].

Following replacement of corticosteroids, levothyroxine, and human somatotropin (0.18 mg/kg/week, subcutaneously, once daily, 6 days per week), growth velocity increased to 10 cm/year (+2.05 S.D) over the first 8 months, more than twice the velocity of previous years (Figure 2(a)). Subsequently, growth velocity was maintained at 9 cm/year for the first two years of treatment and then 4.8 cm/yr for the next three years of treatment. By age of 16 years his height was 5.0 S.D. below the mean, an increase of 1.3 S.D. since initiation of human GH and a total increase of 1.9 S.D. since initiation of Idursulfase. At 17 years, GH was stopped due to concern of scoliosis and kyphosis. Growth velocity was 0 cm/yr in the year following the cessation of GH. Molecular testing of the* PROP1* gene did not identify any disease associated mutations and no alternate cause of GH deficiency was identified.

**Case 2 .**

The second case presented at 7 years of age with typical features of Hunter syndrome. He had undetectable serum iduronate 2-sulfatase activity (Floyd Snyder, Alberta Children's Hospital, Calgary) and a novel c.326G>A mutation in the* IDS* gene was identified (Tracy Stockley, The Hospital for Sick Children, Toronto). Routine cranial imaging revealed a large empty sella and posteriorly displaced pituitary gland (Figure 1(b)). Prior to initiation of Idursulfase, height was −4.8 S.D. and average growth velocity was 1.1 cm/year (−6.3 S.D.). The patient began standard Idursulfase replacement at 12.8 years and showed a marginal improvement to 1.4 cm/year in growth velocity (−6 S.D.; Figure 2(b)).

Serum IGF-1 level was 101 *μ*g/L (226–903 *μ*g/L; normal for bone age: 50–286 *μ*g/L) and thus provocative testing was undertaken. Levodopa/propranolol stimulated GH levels were normal with a peak at sixty minutes of 35.3 *μ*g/L (Normal >8 *μ*g/L). Bone age at chronological age of 13 years 11 months was delayed to 5 years 9 months. The remainder of endocrine testing showed no evidence of anterior or posterior pituitary dysfunction including normal thyroid function and adrenal axis. Since this patient had an intact GH response to stimulation he did not qualify for provincial GH treatment and thus treatment was not undertaken. At age 18 years 8 months, GnRH stimulation testing was performed due to lack of progression of secondary sexual characteristics (6 mL testes, sexual maturity rating stage 2) which revealed peak LH of 16 IU/L and FSH of 13 IU/L at 60 minutes.

Despite his normal GH stimulation testing, the patient requested a trial of GH. Following review by local endocrine section, the managing physician (author Paola Luca) agreed to a trial of GH treatment. Despite his chronological age of 19 years 2 months, his growth plates remained unfused on X-ray. GH treatment was started at a dose of 0.1 mg/kg/week and later increased to 0.3 mg/kg/week. Prior to GH initiation, spine and hip x-rays were negative for scoliosis and avascular necrosis; an echocardiogram was completed before and after GH start and a sleep assessment was performed after GH start. Response to GH was poor with a change in height of 1.7 cm over 9 months and GH was stopped at this time (Figure 2(b)). He did not experience any adverse side effects from the injections.

## 2. Discussion

We contrast 2 cases of Hunter syndrome, both of whom were identified to have significant growth impairment in keeping with the underlying diagnosis of Hunter syndrome and incidentally found to have abnormal pituitary anatomy [3].  Case 1 was found to have an ectopic posterior pituitary and anterior hypopituitarism with GH deficiency, secondary adrenal insufficiency, and tertiary hypothyroidism. Case 2 had a large empty sella, posteriorly located pituitary, and no identified endocrine deficits. Patient 1 was treated with hormone replacement and had a sustained improvement in growth velocity compared to previous years. Patient 2, who produced adequate GH, did not show a response to GH injections. Both patients had persistent, significant compromise to their final adult stature. We contrast these 2 cases primarily to highlight the need for careful endocrine assessment of patients with Hunter syndrome. While previous reports suggest that GH therapy may not be helpful in patients with Hunter syndrome generally, Case 1 documents that panhypopituitarism, the frequency of which remains to be determined, may coexist in some patients. In this subset of Hunter syndrome patients, hormone replacement with corticosteroid, thyroid hormone, and growth hormone may be critically important to prevent endocrine-associated morbidity and, potentially, mortality.

A J-shaped sella turcica is a common finding in Hunter syndrome and is not specific to any single MPS disease [6, 7]. To our knowledge, there have been no published reports describing an ectopic posterior pituitary nor empty sella in Hunter syndrome. Among the MPS syndromes, an empty sella has been reported in two patients with MPS VI (Maroteaux-Lamy syndrome). Only one of these patients was found to have isolated GH deficiency, while the other had intact pituitary functioning [8, 9].

Hunter syndrome is characterized by accumulation of the heparan sulfate and dermatan sulfate due to deficiency of iduronate 2-sulfatase. A number of endocrine organs have been identified to have significant accumulation of this glycosaminoglycans in autopsy studies. These organs include the pancreas, adrenocortical cells of the adrenal, Leydig cells of the testes, follicular epithelial cells of the thyroid, and chromophobe cells of the pituitary [10, 11]. Despite the presence of glycosaminoglycans within the pituitary gland, pituitary function has typically been reported as normal. Toledo and colleagues reported on 3 brothers with Hunter syndrome in whom basal GH levels were within the high-normal range [2]. Responses to GH provocation were normal by insulin tolerance test and lysine-vasopressin stimulation. An absent response to L-dopa was found in the oldest brother. Nelson and Carson reported on one 13-year-old patient who was shown to have an intact GH, adrenal, thyroidal, and gonadal axis response to standard provocative testing [1].

The identification of anterior hypopituitarism in Case 1 is of critical importance. Diagnosis, treatment, and education surrounding central adrenal insufficiency and hypothyroidism may have significant impact on quality of life, morbidity, and mortality. Of note, other than his growth failure, our patient was asymptomatic in spite of being found to have hypopituitarism. He did not have a significant change in symptomatology following hormone replacement, other than an improvement in growth.

The response to GH in Hunter syndrome is mixed; however, despite an adequate response, final height typically remains significantly impaired. Polgreen and Miller published a report documenting GH treatment in two Hunter syndrome subjects [12]. The first met diagnostic criteria for GH deficiency with a peak arginine-clonidine stimulated GH level of 6.6 *μ*g/L. The second had a borderline peak GH level of 9.6 *μ*g/L. Both subjects were treated with low dose GH treatment (0.1 mg/kg/wk) and had good response to GH in the first year of treatment. Patient 1 had an increase in growth velocity to 10.3 cm/year in the first 6 months decreasing to 3.4 cm/year in the following 6 months. Similarly, patient 2 had an increase to 14.2 cm/year and decreased to 7.6 cm/year in the subsequent 6 months. The only adverse event occurred in the second patient, with lower leg pain. Final height was not reported. A longitudinal observational study assessing the effect of GH treatment in 23 Hurler syndrome and Hunter syndrome patients found no difference in growth velocity between those treated with GH versus those untreated [13]. However, those treated with GH deficiency trended towards a higher growth velocity.

Overall, among all MPS syndromes, experience with GH treatment is limited. The largest group reported in the literature is among those with Hurler syndrome (MPS type IH). A retrospective review assessed 8 children treated with GH following hematopoietic cell transplantation [14]. Three of these children had documented GH deficiency (stimulated GH peak < 10 *μ*g/L). Following 1 year of treatment, average growth velocity had a modest increase from 3.5 to 5.2 cm/year. The authors highlighted the need to carefully monitor orthopedic comorbidities in MPS syndromes when administering GH; they reported 4 separate musculoskeletal complications including progression of kyphosis, worsening of scoliosis, worsening of* genu varum*, and new onset slipped capital femoral epiphysis.

It is important to highlight that the majority of published reports on GH use in Hunter syndrome have focused primarily on short term growth response. Very few have documented response to final adult height. While modest gains have been reported, final adult height typically remains significantly impaired. Additionally, in some cases, this may be at the expense of musculoskeletal complications. Ultimately, study and careful thought is required to evaluate the impact of such therapies on patient quality of life. While Case 1 had demonstrated an improvement in height with GH treatment, this was associated with the development of scoliosis and kyphosis.

A clinical conundrum exists in identifying patients with endocrine deficits in Hunter syndrome, particularly GH deficiency. Growth failure is a ubiquitous feature of Hunter syndrome with impaired growth beginning at 4–6 years. By 12 years, virtually all patients fall below the 3rd height percentile [3, 12] and fail to demonstrate a pubertal growth spurt [15]. With institution of enzyme therapy a benefit in height scores has been observed [16]. Both of our patients reported here had a mild improvement in growth velocity following enzyme replacement, but on-going growth failure led to screening endocrine evaluation and subsequent provocative testing.

In our reported patients, screening endocrine tests with IGF-1 were not beneficial as both patients had low results for age while these normalized when corrected for bone age. Based on these 2 patients, neither anthropometric data nor screening investigations were useful in differentiating GH deficiency in Hunter syndrome. Given these observations, we would advocate judicious use of functional endocrine testing in patients with Hunter syndrome. As demonstrated in Case 1, this is particularly important in those with abnormal pituitary imaging studies in whom clinically significant comorbid hypopituitarism may be found.

To conclude, we present 2 unrelated Hunter syndrome patients both evaluated for hypopituitarism following identification of radiographic anomalies of the pituitary. While both cases had similar initial growth patterns and presentations, only Case 1 was identified to have hypopituitarism, with GH deficiency, secondary adrenal insufficiency, and tertiary hypothyroidism.  Case 1 demonstrated a significant response to hormone replacement with a sustained growth response to treatment. Case 2 demonstrated normal pituitary function and did not respond to a trial of GH. These cases highlight the need for careful endocrinological evaluation in children with Hunter syndrome.

## Figures and Tables

**Table 1:** Results of functional endocrine testing in Case 1.

Stimulation test	Time (min)	0	20	30	40	60	120
L-dopa/propranolol	GH (*µ*g/L)	0.1	—	0.1	—	0.1	0.2
1 mcg ACTH	Cortisol (nmol/L)	44	—	149	—	177	—
TRH	TSH (mIU/L)	3.15	28	—	45	43	41
	Prolactin (*µ*g/L)	30	55	—	54	52	48
